# Passive and low-energy strategies to improve sleep thermal comfort and energy resilience during heat waves and cold snaps

**DOI:** 10.1038/s41598-024-62377-5

**Published:** 2024-05-31

**Authors:** Arfa Aijazi, Thomas Parkinson, Hui Zhang, Stefano Schiavon

**Affiliations:** 1 Center for the Built Environment (CBE), University of California, Berkeley, CA USA; 2 School of Architecture, University of Waterloo, Cambridge, ON Canada; 3 Indoor Environmental Quality (IEQ) Lab, The University of Sydney, Sydney, NSW Australia

**Keywords:** Engineering, Civil engineering, Mechanical engineering, Energy science and technology

## Abstract

Sleep is a pillar of human health and wellbeing. In high- and middle-income countries, there is a great reliance on heating, ventilation, and air conditioning systems (HVAC) to control the interior thermal environment in the bedroom. However, these systems are expensive to buy, maintain, and operate while being energy and environmentally intensive—problems that may increase due to climate change. Easily-accessible passive and low-energy strategies, such as fans and electrical heated blankets, address these challenges but their comparative effectiveness for providing comfort in sleep environments has not been studied. We used a thermal manikin to experimentally show that many passive and low-energy strategies are highly effective in supplementing or replacing HVAC systems during sleep. Using passive strategies in combination with low-energy strategies that elevate air movement like ceiling or pedestal fans enhances the cooling effect by three times compared to using fans alone. We extrapolated our experimental findings to estimate heating and cooling effects in two historical case studies: the 2015 Pakistan heat wave and the 2021 Texas power crisis. Passive and low-energy strategies reduced sleep-time heat or cold exposure by 69–91%. The low-energy strategies we tested require one to two orders of magnitude less energy than HVAC systems, and the passive strategies require no energy input. These strategies can also help reduce peak load surges and total energy demand in extreme temperature events. This reduces the need for utility load shedding, which can put individuals at risk of hazardous heat or cold exposure. Our results may serve as a starting point for evidence-based public health guidelines on how individuals can sleep better during heat waves and cold snaps without relying on HVAC.

## Introduction

The quantity and quality of sleep affects human health^1–4^ and cognitive performance^5–7^. The thermal environment, characterized by the dry-bulb air temperature, mean radiant temperature, relative humidity, and air speed, affects sleep quality^8,9^. The strong link between sleep and thermoregulation means both excessively hot and cold thermal conditions have a negative impact on sleep outcomes^10^. Feeling too warm during sleep increases wakefulness and decreases time in shortwave sleep prominent in the initial sleep segments, and rapid eye movement (REM) sleep in the later stages^11^. Feeling too cold during sleep primarily affects the later stages of sleep, where REM is dominant^10^ and significantly affects heart rate variability during sleep^12^ which may contribute to higher frequencies of myocardial infarctions (i.e., heart attacks) in the morning^13^ and in winter^14^. The negative effect can be compensated with bedding insulation, for example, one study reported no significant reduction in sleep quality in ambient temperatures as low as 3 °C due to bed covers maintaining a near constant bed thermal environment^12^.

Despite the importance of the thermal environment to sleep, there are limited building regulations or guidelines that specifically address the design temperature in sleeping environments. Therefore, design practitioners generally assume that the same conditions for thermal comfort during waking hours apply to sleep. In the United Kingdom, the Chartered Institution of Building Services Engineers’ (CIBSE) TM59 Design Methodology for the Assessment of Overheating Risk in Homes recommends that the operative temperature in the bedroom from 10 p.m. to 7 a.m. shall not exceed 26 °C for more than 1% of annual hours^15^. Nicol, who was involved in the standard’s development, later suggests that this criteria may be highly conservative as people sleep comfortably at temperatures of 29–31 °C within their personal bed space^16^. Lomas et al.^17^ also finds that the TM59 criterion suggests a much higher prevalence of overheating than was reported by the English Housing Surveys (EHS). Lomas et al.^18^ recently extended this survey analysis to derive new overheating criteria between 26 and 29 °C.

Public health agencies have a role in communicating evidence-based guidelines on coping with extreme temperatures. Currently, most public health guidance focuses on preventing adverse health effects from overheating^19–22^. With regards to the home and personal adaptations, these guidelines are often limited to reducing use of internal heat gains like ovens and lights, opening windows at night (if safe), using air-conditioning, staying hydrated, and dressing in light clothing. Fewer agencies provide guidelines on preventing adverse health effects from a winter power outage or extreme cold^23,24^. Guidance with regards to the home and personal adaptations focuses on using gas-powered generators with caution and dressing in warm layers. In both cases, there are no specific guidelines on sleeping in extreme temperatures. As a result, popular consumer publications attempt to fill this communication gap, demonstrating public interest in the topic, but their guidelines are often based on reader anecdotes and not on sound scientific research.

Conventional heating, ventilation, and air conditioning (HVAC) systems are a common and effective way to regulate the thermal conditions inside residential buildings. Adoption of residential air conditioning correlates to a 75% reduction in heat-related mortality in the U.S. since 1960^25^. HVAC technologies have high penetration in the United States, where over 95% of homes have some form of space heating and 88% have some form of space cooling^26^. However, space heating and cooling systems are energy intensive and historically represent over 50% of energy end use in residential buildings^27^. An average U.S. home of approximately 170 m^2^ ^26^ will require a 3-ton air conditioner^28^ and a 70,000 Btu/h gas furnace^29^. This equates to a power draw of up to 10 kW and 21 kW for centralized cooling and heating respectively. For comparison, the power rating of a portable or window air conditioning unit can be as high as 4.8 kW^30^ and that of a portable space heater can be up to 1.5 kW^31^, but these are designed for much smaller space volumes, like a single room. In a survey of New York City residents, 91% of respondents with air conditioning at home had it installed in their bedroom^32^. Bedroom air conditioning is especially energy intensive because it typically operates continuously throughout the night. Survey data has recorded this behavior in New York City^32^, Hong Kong^33^, China^34^, and Singapore^35^. This indicates a priority for comfortable sleeping environments in many different contexts.

Yet HVAC systems, even when installed, are cost-prohibitive to operate for many households. Over a quarter of the 123.5 million households in the U.S. report energy insecurity, which may result in leaving the home at uncomfortable temperatures (12.2 million households), receiving a disconnect or delivery stop notice (12.4 million households), or being unable to use heating (5.1 million households) or air-conditioning equipment (6.4 million households)^26^. When taking a broader world view, access to HVAC systems as well as reliable and affordable energy is limited to only a part of the world population. According to the International Energy Agency (IEA)’s report on the future of cooling, of the 2.8 billion people living in the hottest parts of the world, only 8% currently have access to air conditioning^36^.

Climate change further challenges the viability of conventional HVAC systems as a means towards comfortable and healthy sleep in several ways. First, diurnal warming asymmetry^37^ means nighttime temperatures are warming faster than daytime temperatures in much of the world, which could especially be a problem in cooling dominated climates. Atypically warm nighttime temperatures are associated with elevated mortality^38^ and poor sleep quality^39^ particularly among those with limited ability to cope, such as the low-income and elderly persons. Higher nighttime temperatures will increase the energy consumption of existing air conditioners and drive installation of new ones, further exacerbating climate change and urban overheating^40^. Second, the greater frequency and intensity of climate change impacts like heat waves, cold snaps, and wildfires increases the probability of power disruptions. These events compound the disaster^41^ as was seen in British Columbia, Canada and the U.S. Pacific Northwest in summer 2021 and in Texas in Winter 2021^42^. The high energy intensity and low resilience of conventional HVAC systems necessitate an alternate strategy, such as personal comfort systems (PCS) to maintain comfortable and healthy indoor air temperatures during sleep.

PCS are thermal systems that heat and/or cool the individual rather than the entire space and are under the individual’s control^43^. Most research on applications of PCS has focused on increasing thermal comfort and reducing energy consumption in office buildings^44^. However, PCS may be well-suited for sleeping due to the stationary nature of the person. They are cheaper to operate as they require less power (1–100 W) than conventional HVAC systems. Some devices are so efficient that they can be battery operated, making them resilient to utility power interruptions. PCS can also be implemented as part of a strategy to reduce building HVAC energy consumption by extending air temperature set points^45–47^.

A few studies have reviewed the impact of PCS on sleep quality and thermal comfort. Lan et al^48^ found localized cooling of the back and/or head with a hypothermia blanket significantly improved objective and subjective measures of sleep quality in a relatively hot environment (32 °C). Other studies found that head cooling by means of a pillow with a chemical cooling medium improved sleep quality^49^ and decreased the sweat rate^50^, a physiological measure of thermal strain. Increased air movement with fans in a relatively hot environment (30 °C) maintained thermal comfort and sleep quality compared to conventional air-conditioning set to 27 °C^51^. In cold ambient temperatures (5 °C), Song et al.^52^ found a partial-body heating system with a heated electric blanket improved thermal comfort and sleep quality. Okamoto-Mizuno et al.^53^ also found a heated electric blanket to decrease cold stress in a 3 °C environment during sleep.

Fans can be considered a form of PCS and are a relatively common amenity in U.S. homes, with over 70% of households having at least one ceiling fan and over 40% having at least one floor or window fan^26^. Elevated air movement using fans can replace or augment cooling from air conditioning in normal and extreme thermal conditions. The elevated air movement from fans increases thermal comfort at higher air temperatures by accelerating convective and evaporative heat loss from occupants. Other benefits of fans include ease of personal control, improved air distribution, improved perceived air quality, initial HVAC cost savings, and energy savings^54–56^.

Although the power consumption of PCS is significantly less than conventional HVAC systems, both are considered active strategies as they require an energy input. Alternatively, there are many passive adaptations to improve sleep quality that do not require energy input. Examples include change of bedclothes and bed type, and changing posture^16^. In a hot environment, a rope bed, such as the *charpai* in South Asia, may provide more cooling than a conventional mattress. Other behavioral adaptations include migrating to different levels of a building based on the principle of heat rising, i.e., sleeping downstairs or on the floor in the summer and sleeping upstairs in the winter, or even sleeping outside in warm weather to take advantage of radiative sky cooling.

We know that sleep is crucial for human health and wellbeing, and that the thermal environment can affect sleep quality. The current approach of relying on conventional HVAC to control the thermal environment is challenging from an energy, sustainability, and affordability perspective—issues further exacerbated by climate change. We do not know enough about the role that localized interventions like PCS and other personal adaptations can play in improving sleep quality.

## Approach

We used a dry heat loss thermal manikin^57^ in a controlled environmental chamber to evaluate the impact of 11 passive and low-energy strategies on a body’s heat loss in the context of sleeping. These strategies ranged from simple measures such as changing clothing and posture (similar to sleeping) or using a fan, to more advanced products like a hydro-powered mattress pad. We tested heating strategies at an indoor air temperature of 16 °C and cooling strategies at 28 °C. For each strategy, we measured the thermal manikin skin temperature and heat loss at 16 body segments. We then calculated the equivalent temperature, defined as the temperature in which a thermal manikin with realistic skin temperature would lose heat at the same rate as it does in the actual environment, for each of the 16 body parts^57^. The whole-body equivalent temperature is the area-weighted average over all body segments. The difference in equivalent temperature between the thermal manikin with a heating or cooling intervention and the baseline condition gives the heating and cooling effect^68^. While this metric will be conservative for cooling since the thermal manikin cannot measure latent heat loss, such as the evaporative cooling effect from sweating, it still allows us to quantitatively compare the different heating and cooling strategies.

To examine the potential implications of our laboratory findings, we applied our results to two historical case studies: the 2015 Pakistan heat wave and the 2021 Texas power crisis during a winter storm. These represent extreme heat and extreme cold events where conventional HVAC systems were not available either due to lack of access or a multi-day power outage. For both case studies, we modeled both the indoor air temperature of a typical residence during the event and the relationship between our experimentally measured heating and cooling effects of interventions and those predicted indoor air temperatures. We calculated the intensity and duration of hazardous heat or cold exposure during sleep with and without top-performing heating and cooling interventions. During the case study period, we calculated hazardous heat or cold exposure as the difference between the modeled indoor temperature and the World Health Organization’s (WHO) guidelines for indoor minimal risk temperature for adverse health effects^58^. We only considered hazardous heat or cold exposure during sleeping hours, defined as 10 p.m.–7 a.m. per CIBSE TM59. We assume that outside of sleeping hours, individuals would have access to a different set of heating or cooling interventions.

For the 2021 Texas power crisis, we also modeled the impact of coupling low-energy and passive heating interventions with night-time heating setpoint reductions as a way to reduce demand on the electric grid. We modeled the heating intervention and heating setpoint reductions from 10:00 p.m. on February 13 until 7:00 a.m. on February 14, effectively the first night of significant power outages. To estimate the power load of low-energy heating interventions, we assumed four persons in the household, each using the device continuously overnight.

## Results

**Figure 1:**
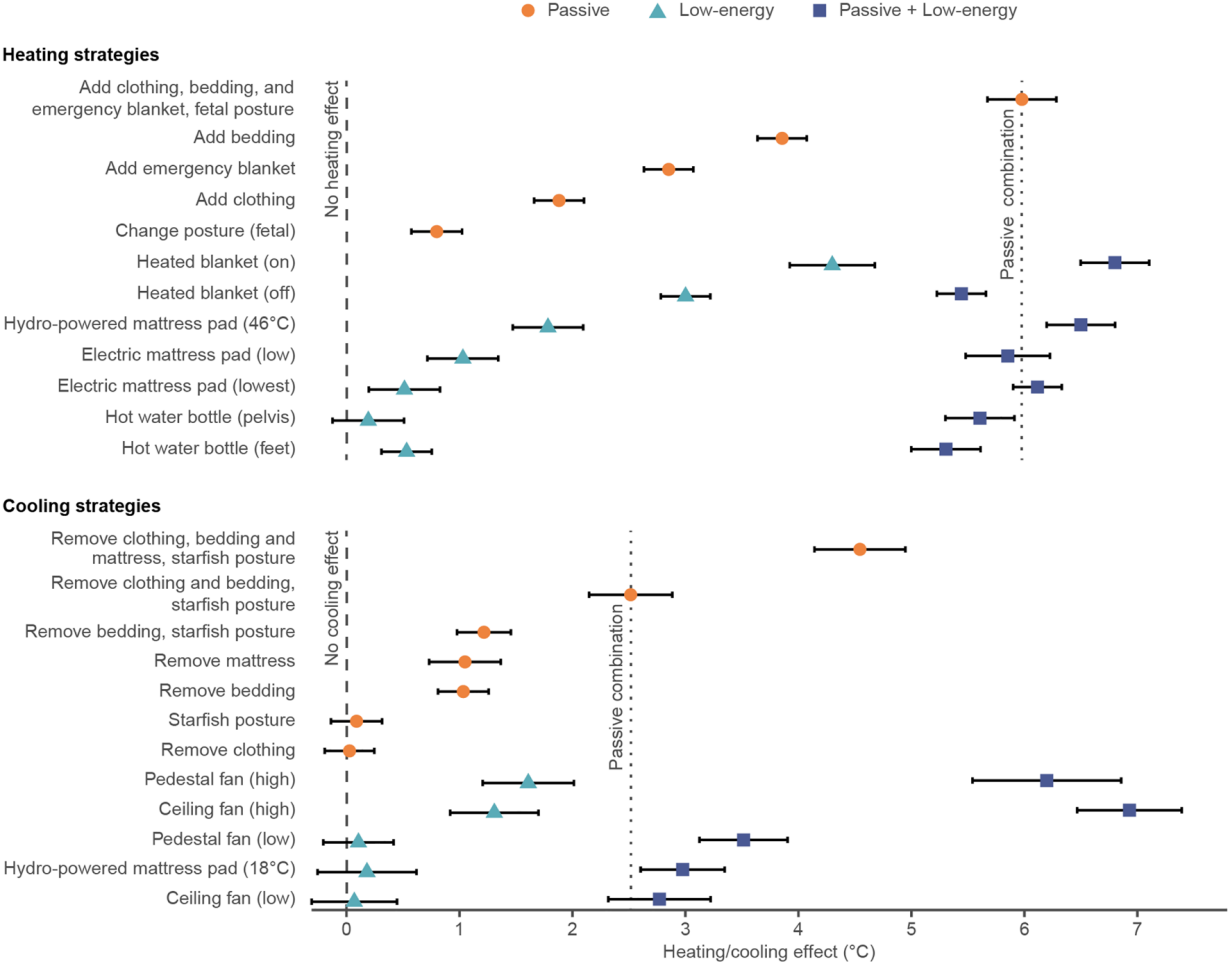
Experimentally determined heating and cooling effect measured by the thermal manikin in a laboratory setting. Results are grouped by passive and low-energy strategies and ordered by decreasing magnitude. The heating or cooling effect is by definition a relative metric, so a value of 0 means there is no heating or cooling effect relative to the baseline and higher values indicate a stronger heating or cooling effect relative to the baseline. Vertical reference lines depict no heating or cooling effect (dashed) and a combination of passive strategies (dotted) for comparison to low-energy strategies. For heating strategies, the selected combination of passive strategies is adding clothing, bedding, and emergency blanket with fetal posture. For cooling strategies, the selected combination of passive strategies is removing clothing and bedding with starfish posture. Error bars represent the 95% confidence interval (CI).

The results of the thermal manikin tests in Fig. 1 show that many of the tested interventions provide substantial heating or cooling effects, meaning that these solutions can be effective at keeping people in a state of thermal comfort while sleeping. While passive personal adaptations provide an effect equivalent to (or exceeding) that of low-energy strategies, the combined effect is particularly effective at helping to offset more extreme bedroom temperatures. As noted previously, the heating and cooling effect is the difference in the thermal manikin’s equivalent temperature between the baseline condition and the heating or cooling intervention.

All the tested passive strategies for heating had a measurable impact in isolation, with the most effective option, adding bedding, having almost a 4 °C heating effect. Among low-energy strategies, the highest effect came from the heated blanket (~ 4.5 °C), followed by the hydro-powered mattress pad (~ 2 °C), and then the electric mattress pad (~ 1 °C). The electric mattress pad was tested at relatively low settings due to limitations with the thermal manikin test procedure, so it may be more effective than what we can show in this study. The hot water bottle had a significantly lower effect, likely because it is a highly localized strategy, and the water temperature was set relatively low (36 °C), per product recommendations to prevent burns. Real users may use significantly hotter water temperatures like up to 100 °C. Combining the passive strategies led to a heating effect of close to 6 °C. The magnitude of the heating effect is similar between passive and low-energy strategies, meaning individuals could select the most appropriate strategy depending on their circumstances. For example, someone with back problems may not be able to sleep in a fetal position but could consider an electric mattress pad for a similar heating effect. An emergency blanket may not be preferred on a day-to-day basis but could be considered under more dire circumstances like a power outage.

On the cooling side, only two of the four passive strategies had a measurable cooling effect in isolation. Removing bedding and removing the mattress had a cooling effect of about 1 °C each, while changing sleeping posture or removing clothing had a negligible effect. The cooling effect increases when different combinations of passive strategies are used. We chose the reference case as the best combination without removing the mattress (2.5 °C), since that is not common in high- and middle-income countries. However, a more ventilated bed type, as is used in some cultures, can be highly effective especially in combination with other passive strategies. For the low-energy strategies, the ceiling and pedestal fan both had a strong cooling effect when operated on the high speed. In the tested conditions, neither fan type generated enough air movement in the low setting. The cooling effect from both fan types is almost three times higher when combined with passive such as minimal clothing and bedding.

**Figure 2:**
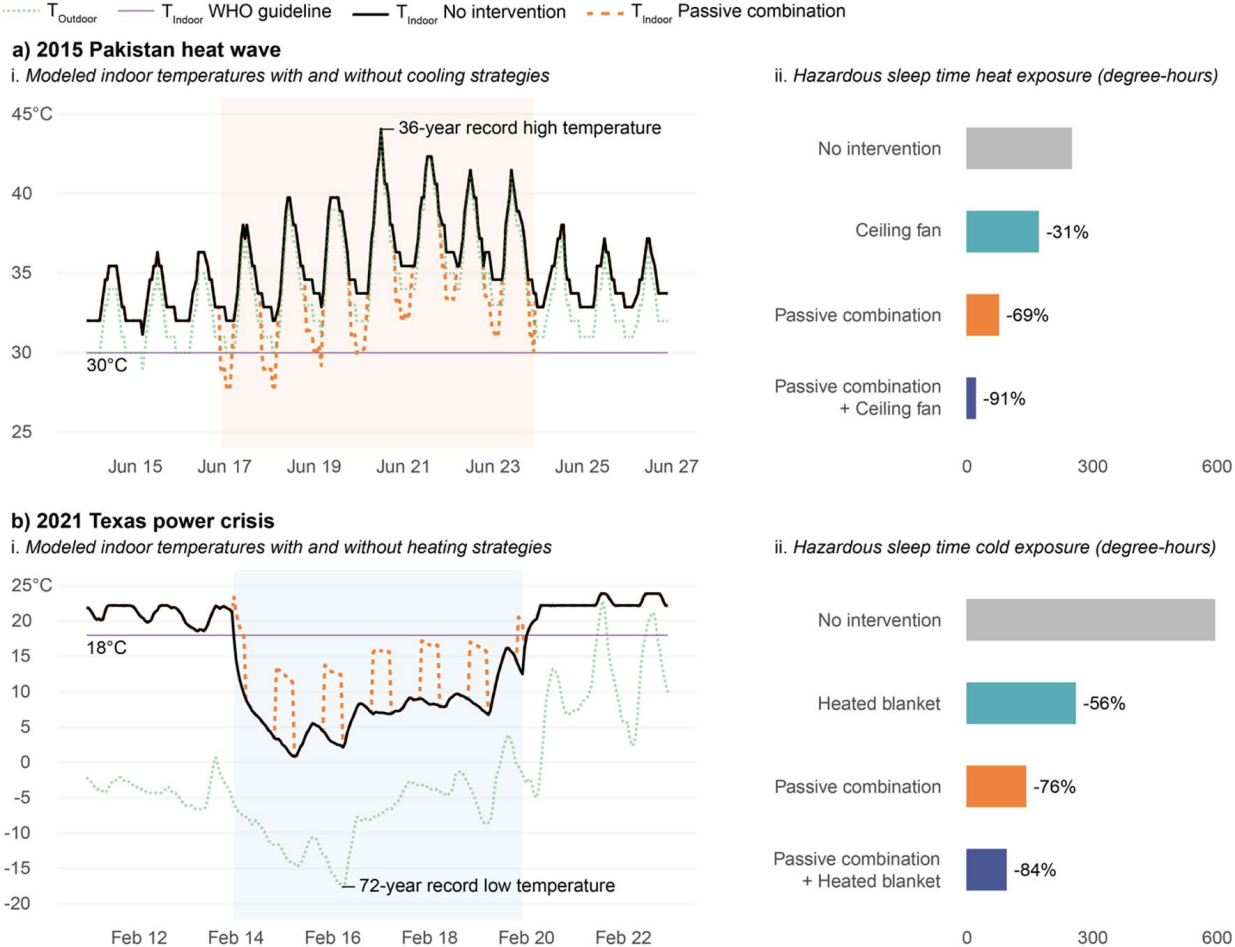
Application of laboratory results of heating and cooling effects to two historical case studies to model sleep-time heat and cold exposure. (**a**) 2015 Pakistan heat wave (June 17–24) and (**b**) 2021 Texas power crisis (February 14–19) as a i. time series of modeled indoor temperatures with and without heating or cooling effects caused by selected interventions and ii. Sleep-time (10 p.m.–7 a.m.) heat or cold exposure based on the World Health Organization (WHO)’s indoor minimal risk temperature guideline. The higher the sleep-time heat/cold exposure the more potentially hazardous the situation is.

The results of the thermal manikin test demonstrate the potential for passive and low-energy solutions for heating and cooling to minimize thermal discomfort in sleeping environments. When these results are applied to historical case studies, the results in Fig. 2 show that a combination of passive and low-energy strategies could reduce sleep-time heat exposure by as much as 91% and cold exposure by as much as 84% during these extreme weather conditions. Relying only on passive strategies, which may be necessitated by a power outage, could still reduce sleep-time heat exposure by 69% and cold exposure by 76%. These reductions in thermal discomfort or stress demonstrate the potential of passive and low-energy strategies to alleviate heat and cold exposure and improve human health and wellbeing.

**Figure 3:**
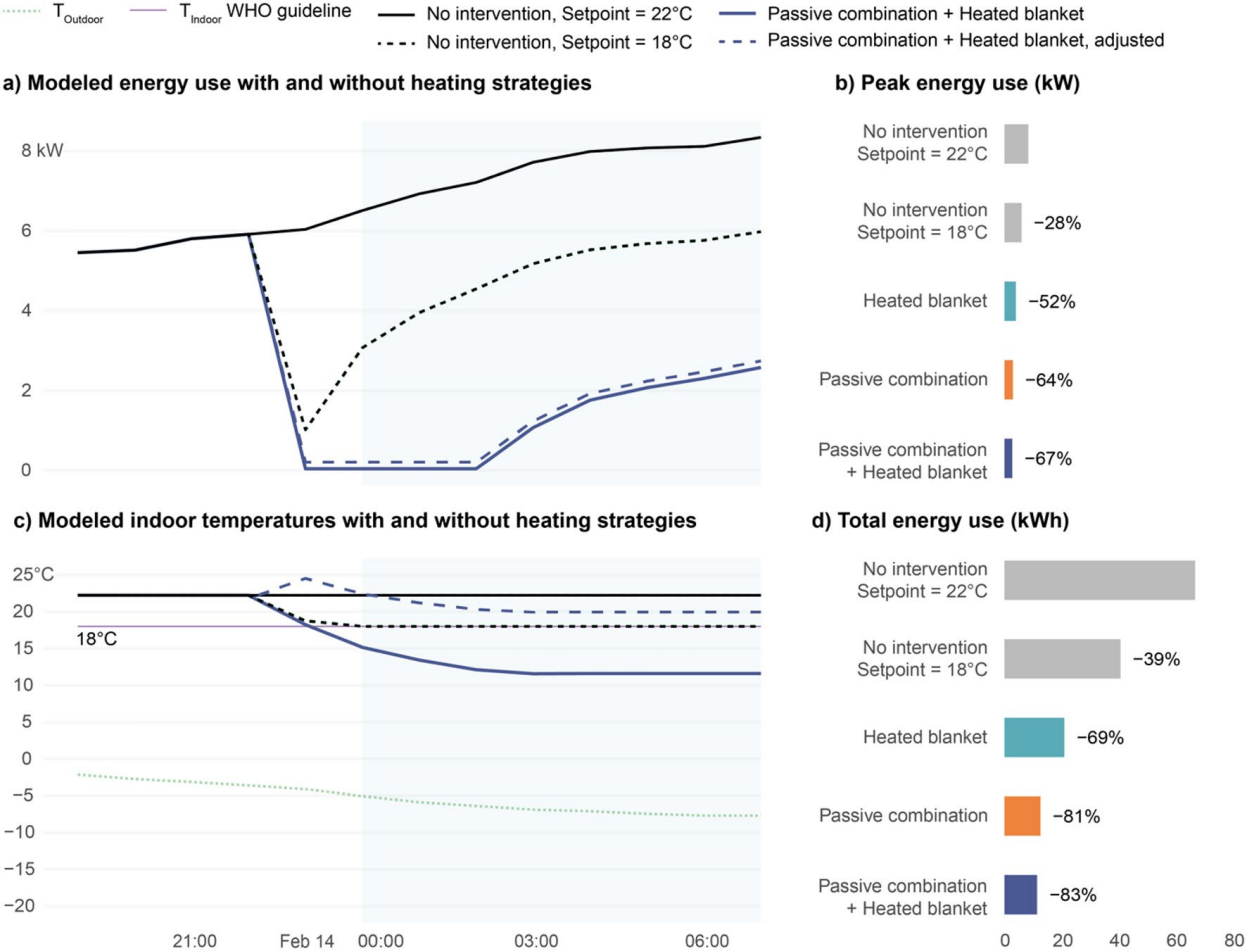
Application of laboratory results of low-energy and passive heating devices to historical case study to model reduced energy loads. (**a**) and (**c**) 2021 Texas power crisis (February 14–19) as a time series of modeled indoor temperatures and energy consumption with and without heating effects caused by selected interventions and (**b**) and (**d**) sleep-time (10 p.m.–7 a.m.) reductions in peak energy use and total energy use overnight from February 13–14. The adjusted energy consumption from the low-energy heating intervention assumes use by four persons continuously overnight.

We also applied the laboratory findings of the manikin tests to the 2021 Texas power crisis case study to model the energy impact of coupling low-energy and passive heating interventions with lower heating set points during sleep-time hours. The original reference energy model for Dallas had a heating setpoint of 22 °C, which exceeds the WHO’s indoor minimal risk temperature guideline of 18 °C. We first modeled the effect of reducing the setpoint to the WHO guideline and then subsequently reduced the setpoint so that the heating effect from the intervention would bring the equivalent temperature to within the WHO recommendation. This resulted in a temperature setpoint of 13.9 °C for a heated blanket, a setpoint of 12.3 °C for the passive combination, and a setpoint of 11.6 °C for the passive combination and heated blanket. While the thermostat setpoint is significantly lower, we note that it is still warmer than the indoor temperatures modeled in the home during the power outage with no intervention, Fig. 2b. The results in Fig. 3 show that reducing the setpoint to the WHO guideline gives a 28% reduction in peak loads and a 39% reduction in total energy use during sleep-time hours. Applying passive strategies and the top performing low-energy heating strategy (a heated blanket), lowers the peak loads by up to 67% and the total energy consumption by up to 83%, effectively double that of just lowering the setpoint alone. While the heated blanket consumes a nominal amount of energy, even the load from four persons is small relative to the energy savings from reducing the thermostat setpoint. The heated blanket is much more efficient as its heat is directed at the person, rather than heating the entire space volume.

## Discussion

Our laboratory results demonstrate that many passive and low-energy strategies can provide a practically relevant heating or cooling effect, making them viable alternatives or supplements to HVAC systems for sleeping environments. In comparison to conventional HVAC systems, low-energy strategies require one or two orders of magnitude less energy, resulting in significantly lower cost. The average cost of household electricity in the United States in September 2023 is $0.16^59^, which is significantly cheaper than other parts of the world^60^. Therefore, a 3-ton air conditioner would cost $14.40 to run overnight (10 p.m.–7 a.m.). The average cost of household natural gas in the United States in September 2023 is $21.85/1000 ft^3^ ^61^. In the U.S. in September 2022, the average heat content of natural gas delivered to end use sectors is 1038 Btu/ft^3^ ^62^. Therefore, a 70,000 Btu/h gas furnace would cost $13.26 to run overnight. Assuming portable heating and cooling options are installed in three bedrooms^63^, three window air conditioning units would cost $20.70 to run overnight and three space heaters would cost $6.48 to run overnight. In comparison, our most effective low-energy cooling (ceiling fan) and heating options (heated blanket) use 18 W and 41 W respectively. Either of these devices would cost mere cents to run overnight.

In extreme events like the 2015 Pakistan heat wave and the 2021 Texas power crisis, load shedding or rolling blackouts by utility providers to manage the surge in demand puts individuals at risk of prolonged exposure to hazardous indoor thermal conditions. Utility providers and emergency planning agencies must consider ways to reduce peak loads during extreme events to avoid more drastic curtailment measures. Our results show that encouraging use of effective passive and low-energy strategies during sleep could help minimize load on energy grids while reducing exposure to extreme indoor conditions. This may take the form of free distribution or subsidies for low-energy devices, particularly to members of vulnerable populations, and public service announcements encouraging use of passive strategies. In this paper, our emphasis is on interventions directly applicable to individuals or their sleeping spaces. There are additional interventions associated with enhancing building quality or landscapes, such as weatherization, shading, natural ventilation, and vegetation. While we acknowledge their potential importance and recommend further exploration, they were not specifically addressed in our study.

While passive and low-energy strategies may already be used in many contexts, there remain barriers to their preferential use over conventional HVAC systems. A study in New York City, United States, shows that passive strategies are less commonly used than other environmental modifications such as turning on the air conditioning or electric fan^32^. There are social and cultural barriers to the use of passive strategies, for example, people may prefer to sleep with a covering, regardless of the indoor air temperature, because it is associated with feelings of safety and security^64^ or for other reasons such as privacy. Likewise, swapping a mattress for a more ventilated bed type may not be a realistic strategy in many parts of the world. While we tested an innerspring mattress, solid foam mattresses, which create warmer conditions, are becoming increasingly common in many parts of the world^65^. Our study suggests the actual heat transfer impact of the mattress may not be as significant as clothing or bedding due to higher contact surface area of those layers compared to the relatively limited contact surface area with the mattress. In reality, a person's surface contact with the mattress will likely be higher than what we modeled in this study due to a real adult weighing approximately 2.9–4 × more^66^ than the thermal manikin and the firmness of a real mattress will decrease over time. Despite this, we would still expect a higher contact surface area with clothing or bedding compared to the mattress.

While our study focuses on the impact of thermal conditions on sleep quality, fans may contribute to other factors related to the overall indoor environmental quality of the bedroom, such as acoustics. Loud environmental noise from vehicular traffic, aircraft, trains, and wind turbines can be disruptive to sleep^67^. These noises are generally loud and intermittent. On the other hand, soft and constant noise, such as that produced by a white noise machine may aid sleep^68^. The noise from fans, like the ceiling and floor fans in this study, is more like the second of these two and if a DC motor is used, the sound is most likely not perceivable, but this is an area for further research.

There are some important limitations in our experimental methodology. First, we tested the heating and cooling interventions at relatively mild conditions due to constraints from the experimental facility and thermal manikin. Moving towards more extreme conditions, we expect that the heating effect would increase linearly with decreasing air temperatures. However, the relationship between the cooling effect and increasing air temperature is nonlinear as convective cooling decreases while evaporative heat loss from sweating (not measured by the manikin) tends to increase. Which leads to the second limitation, the thermal manikin used in this study measures dry heat loss only. Our measurements therefore underestimate the cooling effect by ignoring evaporative cooling from sweating; both passive and active strategies may provide a larger cooling effect than what is reported here. This conservative assumption may hold for populations vulnerable to extreme heat, such as the elderly, who have a reduced ability to sweat^69^. Third, we only considered the whole-body thermal effect and not asymmetric heating or cooling of different body segments. This is more important for localized PCS, such as the mattress pads, pedestal fan, or hot water bottle, as the thermal sensation perceived for individual body segments affects thermal sensation and comfort for the whole body^70^. Fourth, the manikin-based equivalent temperature does not consider human sensation, perception, and other subjective aspects of thermal comfort. Lastly, we assume that someone closer to a state of thermal comfort will have improved sleep quantity and quality, and subsequently other health outcomes.

## Methods

### Experimental facilities

We conducted the study in the controlled environmental chamber (CEC) at the University of California, Berkeley. The chamber measures 5.5 m × 5.5 m × 2.5 m (18 ft × 18 ft × 8 ft 4 in.) and is described in detail by Bauman and Arens^71^ and Arens et al.^72^. Though the CEC’s design resembles a modern office, its mechanical systems provide a high degree of control over the thermal environment. The chamber’s air handling unit (AHU) can control dry-bulb temperature to within 0.2 °C. There are windows on two sides (south and east) with external shades and internal venetian blinds on both windows to control heat gains from solar radiation. An independent HVAC system controls the interior surface temperature of the exterior walls and windows. We set the temperature of interior surfaces to be isothermal with the chamber to ensure mean radiant temperature was the same as the dry-bulb temperature.

### Measuring instruments

We used a dry heat loss thermal manikin to evaluate the effect of different heating and cooling strategies for sleeping environments. A thermal manikin is a heated dummy designed to simulate heat exchange between the human body and its thermal environment^57^. We used a female thermal manikin developed by PT Teknik^73^. The manikin consists of a molded polystyrene shell wound with embedded nickel wire. The manikin is 1.68 m tall and weighs approximately 20 kg^73^. The manikin has 16 independently controlled body segments and measures the sensible heat loss, *Q*_*t*_ [W/m^2^] and the skin temperature *T*_*sk*_ [°C] of each segment. Table 1 lists the surface area and core temperature set point for each body segment.

**Table 1:** Thermal manikin body segments and associated surface area and core temperature setting for comfort mode per the Advanced Berkeley Comfort model^74^.

	Body segment	Surface area (m^2^)	Core temperature (°C)
1	Left foot	0.05	35.1
2	Right foot	0.04	35.1
3	Left lower leg	0.09	35.6
4	Right lower leg	0.09	35.6
5	Left thigh	0.16	35.8
6	Right thigh	0.17	35.8
7	Pelvis	0.17	36.3
8	Head	0.11	36.9
9	Left hand	0.04	35.4
10	Right hand	0.04	35.4
11	Left forearm	0.05	35.5
12	Right forearm	0.05	35.5
13	Left upper arm	0.07	35.8
14	Right upper arm	0.08	35.8
15	Chest	0.14	36.5
16	Back	0.13	36.5
	Total	1.48	36.7

We operated the manikin in “comfort control” mode, which calculates the power supplied to each body segment based on the deep body or core temperature, the measured surface temperature, and the thermal resistance of the skin^75^. This mode of control most realistically represents the temperature distribution of the human body^76^. We set the core temperature of each body segment based on the Advanced Berkeley Comfort model^74^ for a person in in a neutral environment. Prior to data collection, we calibrated the manikin per manufacturer’s instructions to 16 °C and 28 °C^77^.

We monitored the ambient dry-bulb temperature at five locations around the thermal manikin with the Onset HOBO Temperature/Humidity Data Logger (model U12-013 or U12-006) and an air/water/soil temperature sensor (model TMC1-HA) which has a − 40 °C to 100 °C measuring range and 0.25 °C accuracy for temperature. We positioned the temperature probe 0.2 m away from the thermal manikin and elevated it with wood blocks to be vertically centered to the tangent body segment. We calibrated each temperature sensor at three temperatures (15 °C, 25 °C, and 35 °C) using a Polyscience Low-Profile Refrigerated Circulator (model PD7LR-20-A-11B) with a − 20 to 200 °C measuring range and 0.005 °C temperature stability based on a linear regression model for each temperature sensor.

For low-energy interventions, we measured the power consumption using a P3 Kill-A-Watt Plug-In Voltage Tester and Meter (model P4400). This device measures the energy consumption in watts with ± 0.2% accuracy. For contact heating devices, namely the electric and hydro-powered mattress pad and the heated blanket, we measured the power rating with the device in situ with the manikin in baseline condition. For devices that underwent power cycles, meaning the power output was not constant, we measured the average power of completed cycles within ten minutes, which corresponds to the time period of the thermal manikin measurements. The power consumption of the ceiling fan was previously reported in another study^78^. We report the power rating of each device setting in Fig. 6.

### Evaluating the heating and cooling effect

To quantify the heating or cooling effect of different passive and active strategies, we measured the skin temperature and sensible heat loss for each thermal manikin body segment and transformed it into the manikin-based equivalent temperature. The equivalent temperature is defined as the temperature of a uniform enclosure in which a thermal manikin with realistic skin surface temperature would lose heat at the same rate as it would in the actual environment^57^. We calculated the manikin-based equivalent temperature, ${T}_{eq}$ [°C], as follows in Eq. (1), where ${T}_{sk}$ [°C] is the skin temperature, ${Q}_{t}$ is the sensible heat loss [W/m^2^], and ${h}_{cal}$ is the dry heat transfer coefficient [W/m^2^ °C], which we calculated from a nude manikin (as described in a subsequent section and summarized for each body segment in Table 2), and $i$ is the body segment. The whole-body equivalent temperature is the area-weighted average over all body segments.

**Table 2:** Dry-heat transfer coefficient of each body segment of the thermal manikin.

Body segment	16 °C		28 °C	
	Log	Fetal	Log	Starfish
Back	7.8	7.7	7.2	6.8
Chest	7.8	6.9	6.9	6.0
Head	5.1	5.3	4.6	4.5
Left foot	8.5	8.3	7.0	7.9
Left forearm	8.5	8.7	7.7	8.1
Left hand	8.9	9.0	8.3	8.4
Left lower leg	8.5	8.2	7.7	8.1
Left thigh	7.6	7.4	7.3	7.6
Left upper arm	7.9	8.0	7.5	7.9
Pelvis	8.2	7.8	7.7	6.9
Right foot	8.7	8.1	7.6	7.9
Right forearm	9.1	8.0	8.1	7.9
Right hand	9.2	8.5	8.4	8.1
Right lower leg	8.7	8.2	7.9	8.1
Right thigh	8.0	7.7	7.5	7.4
Right upper arm	8.5	7.7	7.4	7.4

1 $${T}_{eq,i}={T}_{sk,i}-\frac{{Q}_{t,i}}{{h}_{cal,i}}$$

The absolute value of the difference in the manikin-based equivalent temperature relative to a baseline condition gives the heating or cooling effect of the strategy, as shown in Eq. (2). This approach is similar to prior studies measuring the heating effect of personal heaters^31^ or the cooling effect of elevated air movement^79,80^.

2 $${\Delta T}_{eq}=\left|{T}_{eq,\,\,heating\,\,or\,\,cooling\,\,strategy}-{T}_{eq, \;Baseline}\right|$$

**Figure 4:**
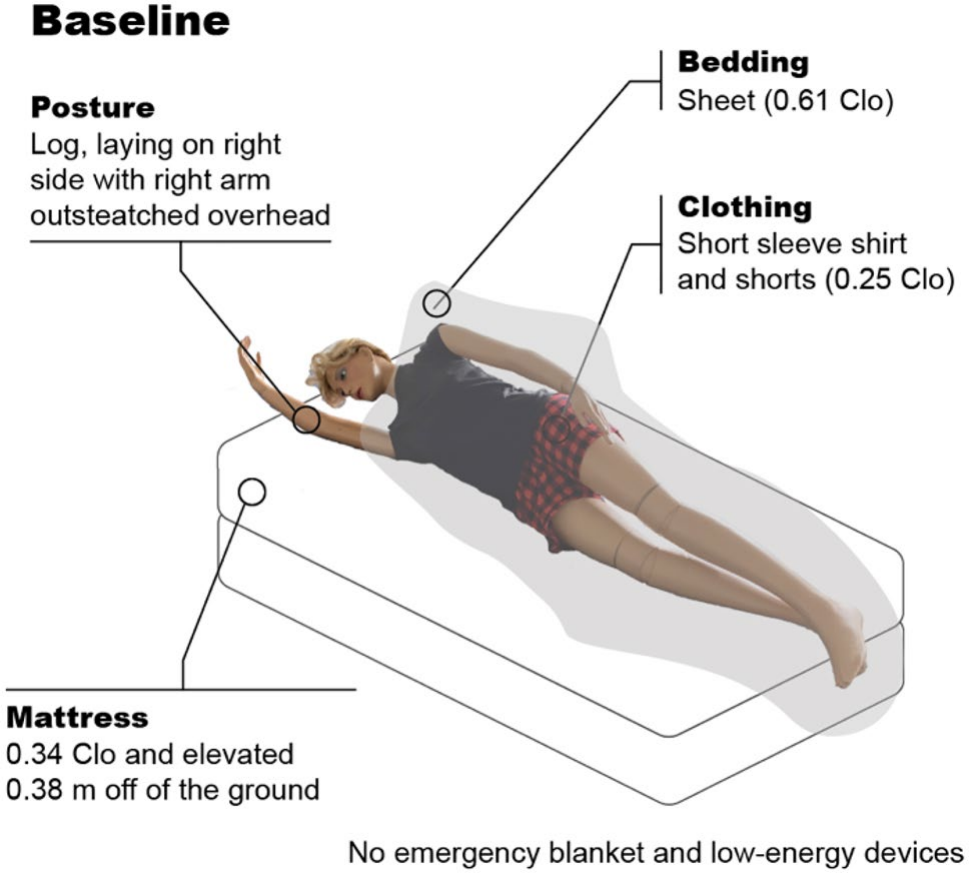
Baseline condition. The baseline condition (thermal manikin posture and clothing, bedding, and mattress) is used to calculate the difference in manikin-based equivalent temperature as shown in Eq. (2).

We defined the baseline condition, illustrated in Fig. 4, as a thermal manikin in the same ambient temperature with light clothing (0.25 Clo), a sheet (0.61 Clo) covering from below the shoulders, laying in log posture (i.e. laying on the right side with one arm outstretched) on a conventional mattress (0.34 Clo) with no emergency blanket or low-energy heating or cooling system.

### Experimental conditions

We measured performance of heating and cooling strategies at 16 °C and 28 °C respectively. The lower temperature was constrained by the CEC’s setpoint limits, which are intended for studies of thermal comfort. The upper temperature was constrained by the thermal manikin, which can only measure dry-heat loss and not heat gain. Relative humidity does not affect thermal manikin measurements and therefore we did not measure or control it.

**Figure 5:**
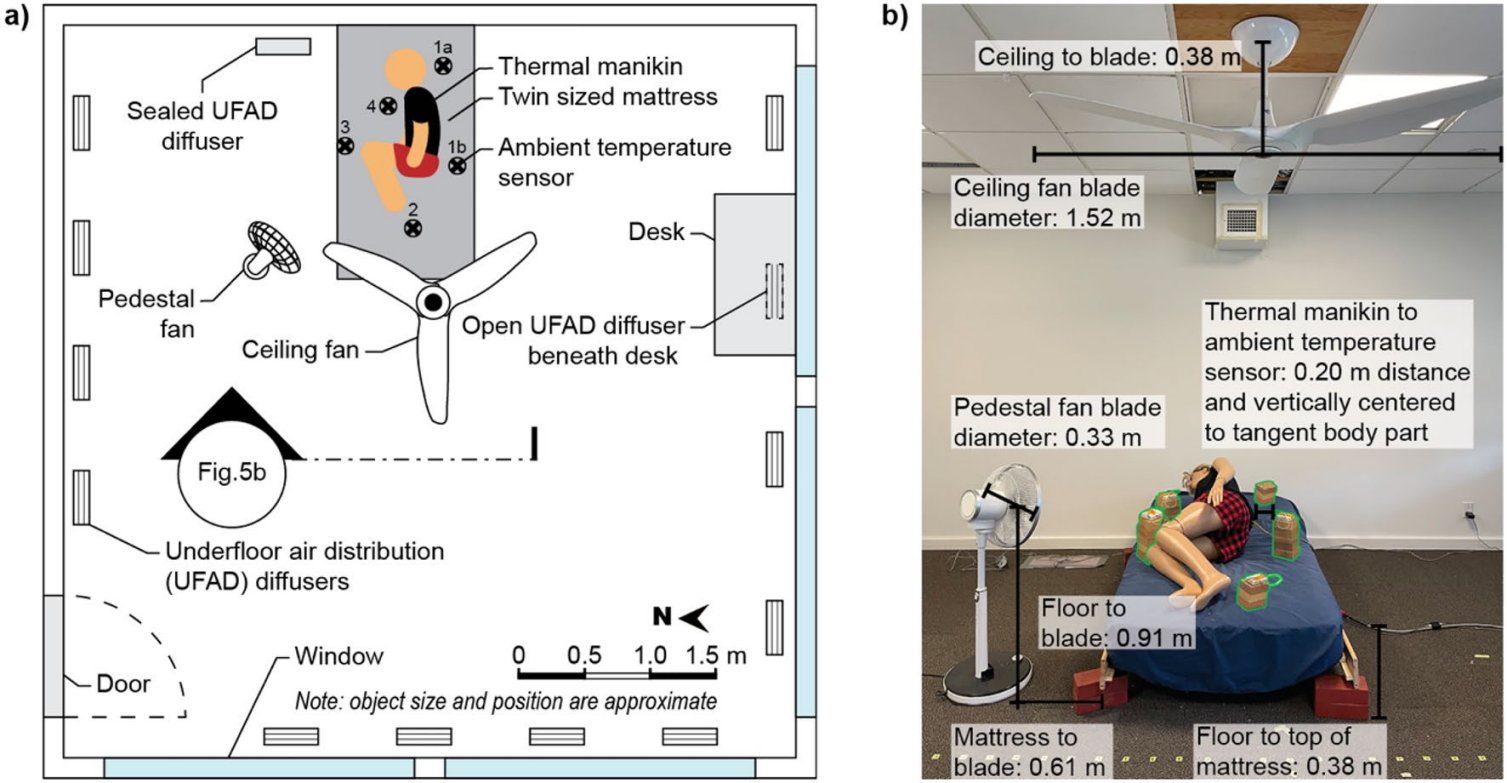
Experimental setup. Plan view of controlled environmental chamber (CEC) and experimental setup; (**b**) Section view of CEC setup prior to experiment with annotations. Ambient temperature sensors (green outline) were added for clarity. Ambient temperature sensors were present during experimental run. (**a**) Contains an adaptation of “sleep” by Gan Khoon Lay from Noun Project, used under CC BY 3.0.

**Figure 6:**
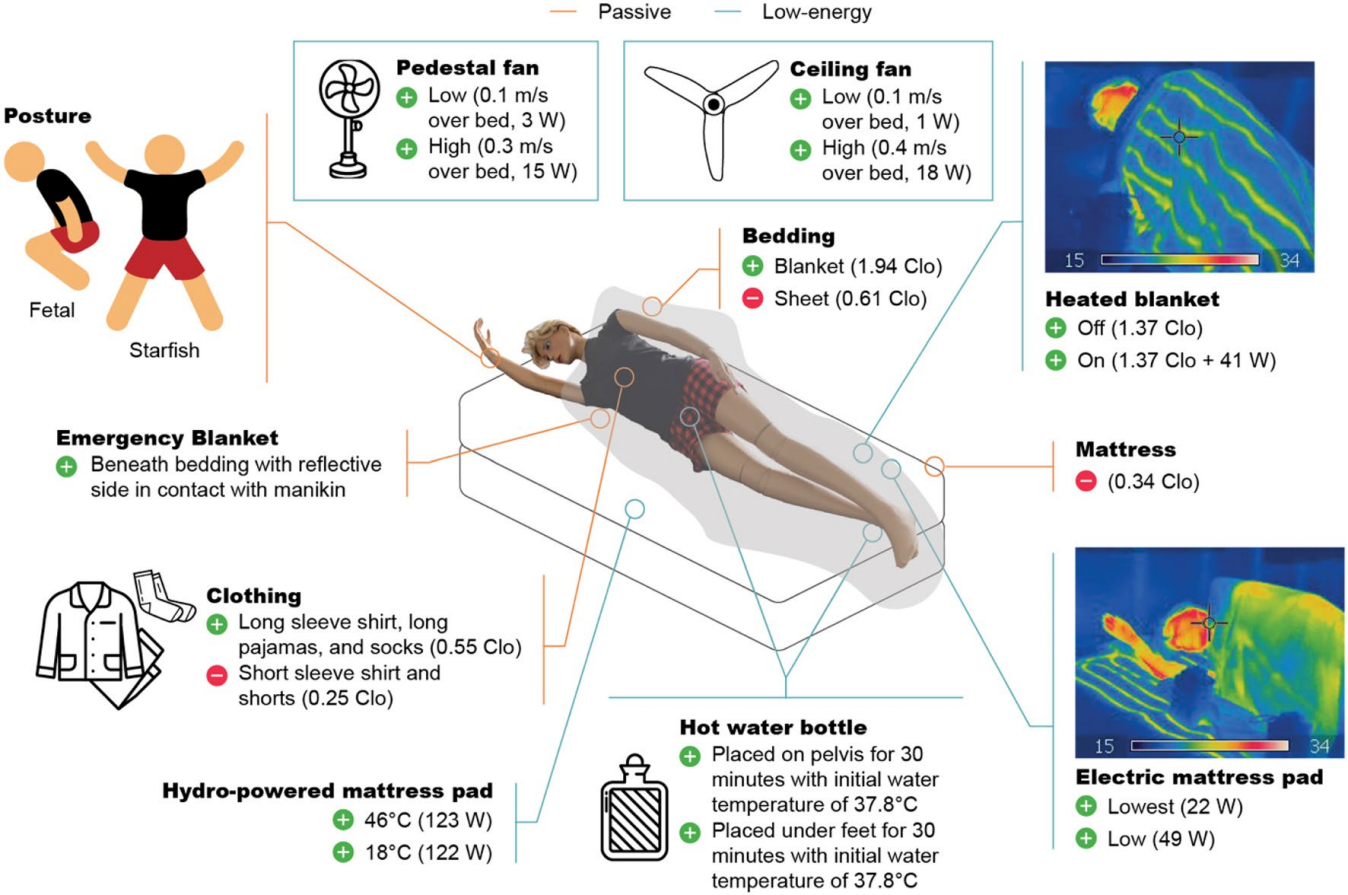
Experimental conditions. Composite illustration of experimental conditions representing a variety of passive and low-energy heating and cooling strategies. Figure contains the following third-party material from Noun Project, used under CC BY 3.0: “Hot Water Bottle” by Icongeek26, “Pajamas” by Jino, “socks” by Lucas Helle, and “pedestal fan” by Atif Arshad as well as adaptations of “Plus” and “Decline Less” by naim and “sleep” (fetal posture) and “sleep” (starfish posture) by Gan Khoon Lay.

Figure 5 shows a diagram of the experimental set up in the CEC. We oriented the head of the bed against the wall, as is typical in a bedroom, where it is away from windows and floor registers. We sealed the floor register nearest to the bed to avoid disrupting the manikin’s thermal plume. We measured the heating or cooling effect of a variety of passive and low-energy strategies depicted in Fig. 6 and describe each strategy in greater detail below.

### Clothing and bedding insulation

Clothing and bedding affect heat transfer via conduction by providing thermal resistance and radiation and convection by trapping a layer of still air between the fabric and skin. The baseline clothing ensemble (“Light”) consists of a cotton short-sleeve t-shirt and cotton shorts (0.25 Clo). The “Heavy” clothing ensemble consists of a polyester long-sleeve button front pajama shirt, long pants, and socks (0.55 Clo). The baseline bedding (“Light”) consists of a cotton U.S. standard twin sized bed sheet (0.61 Clo). The “Heavy” bedding consists of the cotton sheet with the addition of a polyester twin sized blanket (1.94 Clo). We smoothed out clothing and bedding so that it conformed to the manikin body to minimize trapped air between fabric layers. While this study focused on dry heat transfer, it’s worth noting that clothing and bedding with the same insulation level could perform differently with regards to latent heat transfer, like the absorption, diffusion, and evaporation of sweat. Common bedding materials like cotton, linen, or polyester, have different hygroscopicity, wicking ability and permeability.

### Posture

Change of posture is a common physiological response to discomfort during sleep. The change in surface area in contact with the bed versus other body segments can significantly effect heat loss^16^. We considered three postures as part of this study. In the baseline “Log” posture, the manikin is on its right side with the right arm outstretched by the head and the left arm and legs extended straight. In the “Fetal” posture the manikin is on its right side with the left and right arm extended straight and the legs bent towards the chest. The manikin’s rigidity, such as lack of elbow joint, meant we could not achieve a true fetal posture. In the “Starfish” posture the manikin is on its back with both arms outstretched by the head and both legs outstretched.

### Emergency blanket

An emergency blanket, also known as a space blanket or Mylar^®^, blanket, is a lightweight blanket made of heat-reflective, thin, plastic sheathing. We used an emergency blanket manufactured by Survive Outdoors Longer (SOL) made of vacuum-metalized polyethylene that weighs 0.08 kg^81^. First responders often deploy these blankets in emergency situations to prevent or counter hyperthermia. Emergency blankets reduce heat loss by several mechanisms. The air and watertight foil reduces heat loss through convection and evaporation, and the reflective surface reduces heat loss from thermal radiation. Emergency blankets may also be used in conjunction with other bedding to reduce heat loss by conduction. Emergency blankets are inexpensive and commercially available, making them a highly feasible intervention for cold thermal emergencies. We placed the emergency blanket between the thermal manikin and any bedding layer. Like clothing and bedding, we smoothed out the emergency blanket so that it conformed to the thermal manikin body to minimize trapped air.

### Bed type

Modern mattresses are highly insulating (0.34 Clo), so we considered the cooling effect of removing the mattress and laying the manikin directly on the wooden slat bed frame. This type of bed is like a rope bed traditionally used in hot environments, such as the *charpai* in South Asia. We elevated the bed frame so that the manikin was at the same height (0.32 m) as test conditions with the mattress.

### Hydro-powered mattress pad

A hydro-powered mattress pad consists of silicone tubing integrated into a fabric and a control unit that circulates conditioned water. Like an electric mattress pad, a hydro-powered mattress pad is placed below the bottom bed sheet. We used the Cube Sleep System by ChiliSleep which has a temperature range of 13–46 °C subject to environmental conditions. We operated the hydro-powered mattress pad at 18 °C under cooling mode and 46 °C under heating mode. In preliminary testing, we found the hydro-powered mattress pad was unable to sustain temperatures below 18 °C at an ambient temperature of 28 °C.

### Electric mattress pad and heated blanket

An electric mattress pad and heated blanket both consist of an insulated wire or heating element inserted into a fabric that heats up when powered. The difference between these two devices lies in their placement. An electric mattress pad is placed above the mattress and below the bottom bed sheet (below a person) while a heated blanket is placed over the top bed sheet (above a person). We used a SunBeam electric pad under two settings: 1/10 which we describe as “Lowest” and 3/10 which we describe as “Low”. We did not test the electric mattress pad under higher settings because the thermal manikin is unable to measure power when in a state of heat gain. We used a SunBeam heated blanket with both the heated blanket turned “off” and “on” to separate the effect of the electric heated element and the additional insulation. When on, we set the heated blanket to the highest setting: 5/5.

### Hot water bottle

A hot water bottle is a sealed vessel filled with hot water and used to provide warmth in bed or apply heat to specific body segments for pain relief. We used a rubber hot water in a conventional square shape with a capacity of 1.25 L. Immediately prior to each experimental run, we filled the hot water bottle with 1 L of water heated to 37.8 °C (100°F), per manufacturer recommendations to prevent burns. We analyzed thermal manikin data starting from 30 min after hot water bottle placement based on the thermal stability of hot water bottle. We tested the heating effect of a hot water bottle at the feet and pelvis, which are both common locations for hot water bottle use.

### Pedestal fan and ceiling fan

**Figure 7:**
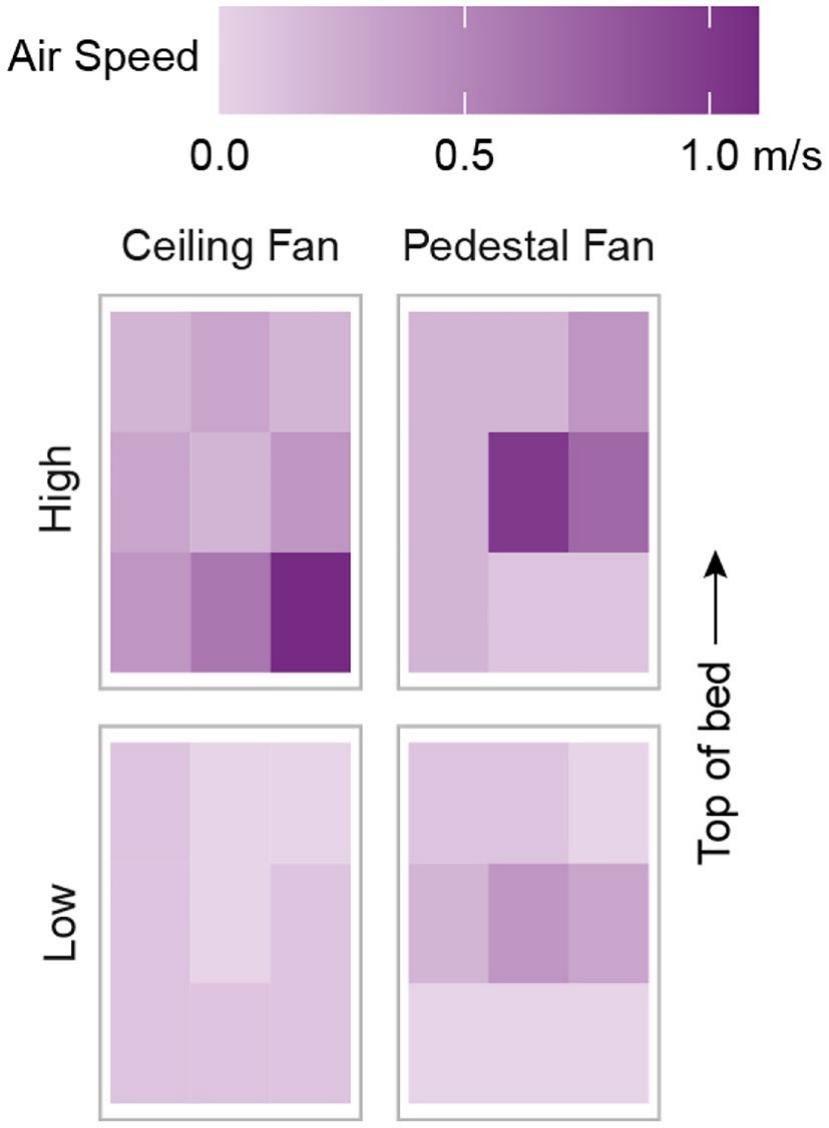
Air speed over the bed. Spatial distribution of average air speed generated by the ceiling and pedestal fans across the bed as measured by a handheld anemometer 0.3 m above the mattress for the low- and high-speed settings.

We tested the cooling effect of a ceiling fan positioned 0.38 m from the ceiling and a pedestal fan positioned 0.61 m from the base of the bed. We tested both fan types at their highest and lowest speed setting. We recorded the air speed four times in a continuous three-minute interval. Figure 7 shows the spatial distribution of average air speed across the bed as measured by a handheld anemometer (TSI VelociCalc Air Velocity Meter Model 8347) at a height of 0.3 m (1 ft) above the mattress. The spatially averaged air speed over the bed for the ceiling fan and pedestal fan was 0.4 m/s and 0.3 m/s on the high setting and 0.1 m/s and 0.2 m/s on the low setting respectively.

### Experimental procedure

We carried out testing after the CEC ambient temperature had equilibrated to within 0.2 °C of the target temperature based on the level of accuracy in the setpoint temperature^72^. We recorded the surface temperature and power consumption of each body segment for ten minutes after reaching steady-state conditions. We defined steady state conditions as when the time-averaged surface temperature difference of each body segment changed less than 0.05 °C in the preceding ten minutes^80^. When starting measurements with a cold thermal manikin, we allowed for at least three hours of warmup time^82^. We randomized the order of experimental trials within twelve blocks of the same chamber temperature setpoint. This is due to the amount of time needed to transition the chamber from one temperature condition to another. We allowed for at least 30 min between experimental runs, due to the time constant of the mattress as measured in preliminary studies.

### Dry-heat transfer coefficient to free convection

We obtained the dry-heat transfer coefficient due to free convection, ${h}_{cal, i}$, for each body segment by calibrating the thermal manikin in a uniform thermal environment i.e. air temperature equal to the mean radiant temperature and air speed less than 0.06 m/s. In this condition, the room air temperature is equal to the equivalent temperature, ${T}_{eq}$, defined in Eq. (1). We performed the calibration at two ambient air temperatures (16 °C and 28 °C) and two postures (log, and fetal at 16 °C and starfish at 28 °C). During the calibration, the thermal manikin was nude and lay directly on the wooden slat bed frame i.e., no mattress. The thermal manikin did not have any bedding, an emergency blanket, or active systems. The results in Table 2 show ${h}_{cal, i}$ for each body segment. This value combined with the thermal manikin skin temperature and heat loss as shown in Eq. (1) allow us to calculate the equivalent temperature.

### Error and uncertainty

We analyzed the data in accordance with the ISO guideline for the expression of uncertainty in measurement^83^. We calculated the combined standard uncertainty by considering equipment intrinsic uncertainty, equipment measurement uncertainty, measurement stability during steady state, and repeated trials of calibration, baseline conditions, and select experimental conditions in accordance with Bell^84^. When presented, we indicate the uncertainty with error bars with a confidence level of 95% (coverage factor of 2).

### Case studies

We applied the laboratory findings to two historical case studies: the 2015 Pakistan heat wave and the 2021 Texas power crisis. In both cases, climate change contributed to the unprecedented weather events^85,86^. The two case studies also represent very different contexts in terms of conventional HVAC availability and construction typology. We assumed conventional HVAC systems were not available either due to lack of access (Pakistan) or multi-day power outage (Texas). For each case study, we modeled the indoor air temperature and the heating and cooling effect as a function of that temperature. We then calculated heat or cold exposure during sleep-time, defined as 10 p.m.–7 a.m. based on CIBSE TM59^15^, according to guidelines from the WHO for the indoor minimal risk temperature for adverse health effects^58^.

### 2015 Pakistan heat wave

In June 2015, Sindh Province in southern Pakistan experienced a severe heat wave with temperatures as high as 49 °C. Overall the heat wave claimed at least 2000 lives^87^. One factor contributing to the high death toll was widespread power outages leaving individuals without access to air conditioners, fans, and water pumps. Power outages aside, less than 5% of the overall Pakistani population had access to AC in 2010^88^.

To quantify the potential impact of passive and low-energy strategies in alleviating heat exposure, we modeled a multi-family residence in Karachi, Pakistan, the most populous city in Pakistan. Karachi is one of the most densely populated cities in South Asia and the limited land resources have shaped a high-density urban morphology^89^, hence the suitability of modeling this type of residence. This construction typology may place inhabitants at higher risk of heat exposure due to more limited opportunities for natural ventilation^17^.

We obtained outdoor dry-bulb temperature and relative humidity data for June 2015 from the Jinnah International Airport weather station through Visual Crossing, a third-party interface for publicly available weather data^90^. We estimated indoor temperature and mean radiant temperature as a function of the outdoor temperature based on a linear regression model of field measurements in the ASHRAE Global Thermal Comfort Database II v 2.1^91^. For the linear interpolation, we used a subset of the full database consisting of 1728 observations of coincident indoor air temperature, mean radiant temperature, and outdoor temperature from approximately 50 naturally ventilated multi-family buildings in Ahmedabad, India. This was the closest construction typology and geographic location to Karachi. Generally, the indoor temperature is a few degrees warmer than the outdoor temperature. We assumed the inside and outside air had the same absolute humidity and recalculated the relative humidity from the saturation vapor pressure of the modeled indoor temperature.

Our experimentally measured cooling effect only accounts for dry-heat loss and not evaporative heat loss. Therefore, we modeled the cooling effect of passive and low-energy strategies with the Standard Effective Temperature (SET) based on the 2-Node Model by Gagge et al.^92^ as implemented in the comf package in the R programming language^93^. The SET model requires six input parameters: indoor air temperature, mean radiant temperature, relative humidity, air velocity, metabolic rate, and clothing insulation. We assumed the mean radiant temperature is equivalent to the indoor air temperatured^94^. For still air, we assumed an air velocity of 0.2 m/s^95^. For cases with elevated air movement, we used the spatially averaged air speed over the bed listed in Table 2. We set the metabolic rate to 0.7 met for a sleeping person^94^ and the clothing insulation as the sum of the mattress, bedding, and clothing from the laboratory experimental condition.

The difference between the modeled SET for the baseline condition and any cooling intervention gives the cooling effect. We then subtracted this cooling effect from the calculated indoor air temperature to compute the sleep-time heat exposure. We considered midnight June 17 until midnight June 24 as the dates of the heat wave for our heat exposure analysis. WHO acknowledges that the minimal risk temperature for heat-related exposure requires further research and provides conditional recommendations based on the climate region. We selected an indoor minimal risk temperature of 30 °C, which WHO provides as an example for Thailand, the closest match to Pakistan in terms of climate (tropical/subtropical) and AC penetration rates below 25%^88,96^.

### 2021 Texas power crisis

On February 13, 2021, a major blizzard and ice storm named Winter Storm Uri^97^ moved across the Southern United States, causing record low temperatures of − 19 °C in northeastern Texas. The storm triggered a major infrastructure failure across the state due to a lack of equipment winterization and a surge in electrical demand from the low temperatures. The state’s electric grid operator reported significant power generation outages from February 14 to 20^42^, leaving millions of homes and businesses without power^98^.

We used the 2021 Texas power crisis as a case study to quantify the potential impact of passive and low-energy strategies to mitigate cold exposure. According to the U.S. Energy Information Agency, 61% of Texas homes rely on electricity as their primary heating source^99^. The American Council for an Energy-Efficient Economy's 2022 State Scorecard ranks Texas 29 out of the 50 U.S. states in terms of policy and program efforts to pursue energy efficiency^100^. In the Building Energy Efficiency category, Texas scored 4.5 out of 12 possible points, suggesting that Texas homes may lack sufficient insulation. Consequently, the winter storm power outages left many Texans with frigid temperatures inside their homes, contributing to the 246 deaths attributed to the winter storm and power outages^101^. We used EnergyPlus v. 22.2.0^102^ to model indoor air temperature in a single-family home with a slab-on-grade foundation based on historical trends for building permit data^103^ and typical constructional practices in Texas^104^. Using energy simulation software more accurately represents the building’s thermal response to the power outage, which would lag due to material heat capacity. We selected Dallas, Texas’s third most populous urban area, as a representative city due to the unprecedentedly low temperatures in that region of that state.

We used the residential prototype building model developed by the Pacific Northwest National Laboratory (PNNL)^105–107^ in the simulation. We selected the single-family, climate zone 3A, electrical resistance heating, slab foundation, International Energy Conservation Code (IECC) 2015 energy model. We made the following modifications to the reference model: updated the file version from EnergyPlus v. 9.5 to EnergyPlus v. 22.2.0 using the EnergyPlus auxiliary preprocessing program IDFVersionEditor, replaced the existing design day data objects with those for Dallas/Fort Worth International Airport, and changed the schedules of all electrical equipment and HVAC to be unavailable from midnight February 14 until midnight February 20, based on net generator outages reported by the state’s electrical utility operator, ERCOT^42^.

We created a custom historical EnergyPlus weather file to use for the simulation. We obtained the hourly dry-bulb temperature, dew point temperature, relative humidity, seal level pressure, global horizontal radiation, wind direction, wind speed, opaque sky cover, visibility, snow depth, and rain quantity for February 2021 from the Dallas/Fort Worth International Airport weather station through Visual Crossing^90^. We converted the sea level pressure to atmospheric pressure based on the dry-bulb temperature and an elevation of 171 m (weather station elevation). We used the EnergyPlus auxiliary preprocessing program WeatherConverter to split the global horizontal radiation into direct and diffuse horizontal radiation components. We ran the simulation for the entire month of February to ensure an adequate initialization period.

To approximate the relationship between our experimentally measured heating effect and indoor air temperature, we assumed the heat transfer coefficient between the person and the environment would remain constant, as represented in Eq. (3), where ${Q}_{t}$ is the sensible heat loss [W/m^2^], $h$ is the dry heat transfer coefficient [W/m^2^ °C], ${T}_{sk}$ [°C] is the skin temperature, and ${T}_{a}$ [°C] is the indoor air temperature. At our experimental conditions, ${Q}_{t}$ and ${T}_{sk}$ come from the thermal manikin and ${T}_{a}$ from the ambient temperature sensors.

3 $${Q}_{t}=h\left({T}_{sk}-{T}_{a}\right)$$

From the 2-Node Model by Gagge et al.^92^, the skin temperature varies by less than 2 °C over a 30 °C dry-bulb temperature range, so we approximated it as a constant over the range of modeled indoor air temperatures. We calculated the heat transfer coefficient at an indoor air temperature of 16 °C based on the measured skin temperature and power supplied to each thermal manikin body segment. Holding the heat transfer coefficient and skin temperature, we then approximated the power needed for new indoor air temperatures to calculate a new equivalent temperature. The difference in equivalent temperature between the baseline and any heating intervention gives the heating effect. We then added this heating effect from the modeled indoor air temperature to compute the sleep-time cold exposure based on WHO’s recommendation for minimal risk temperature of 18 °C during the cold season in temperate and colder climates^58^. We considered February 14–20, 2021, as the dates of the power outage for our cold exposure analysis.

We next modeled the impact of coupling low-energy and passive strategies with reduced heating set points. The original reference energy model for Dallas has a heating setpoint of 22 °C, which exceeds WHO’s indoor minimal risk temperature guideline of 18 °C. We first modeled the effect of reducing the setpoint to the WHO guideline (18 °C) and then subsequently reduced the setpoint so that the heating effect from the heating intervention would bring the equivalent temperature to within the WHO guideline. To estimate the different setpoint temperatures for the modelling exercise, we calculated each intervention's heating effect at 18 °C and then subtracted that amount from 18 °C. The reduced setpoint was applied in the simulation at 10:00 p.m. on February 13 and ended at 7:00 a.m. on February 14, which corresponds to the first overnight period during the 2021 Texas power crisis. As before, we assumed occupants would have other heating measures available after 7:00 a.m during wake hours. Analysis of the simulated peak energy and total energy focused on the sleep-time hours only (10:00 p.m.–7:00 a.m.). To estimate the energy consumption from low-energy interventions, we assumed four people would use the device continuously overnight.

## Data Availability

All data and analysis code is provided on GitHub at: https://github.com/anaijazi/thermalManikinSleep.
